# The Influence of Different Oregano Species on the Antioxidant Activity Determined Using HPLC Postcolumn DPPH Method and Anticancer Activity of Carvacrol and Rosmarinic Acid

**DOI:** 10.1155/2017/1681392

**Published:** 2017-10-18

**Authors:** Juste Baranauskaite, Asta Kubiliene, Mindaugas Marksa, Vilma Petrikaite, Konradas Vitkevičius, Algirdas Baranauskas, Jurga Bernatoniene

**Affiliations:** ^1^Department of Drugs Technology and Social Pharmacy, Lithuanian University of Health Sciences, Medical Academy, Kaunas, Lithuania; ^2^Department of Analytical and Toxicological Chemistry, Lithuanian University of Health Sciences, Medical Academy, Kaunas, Lithuania; ^3^Department of Drug Chemistry, Lithuanian University of Health Sciences, Medical Academy, Kaunas, Lithuania

## Abstract

The aim of this study was to evaluate concentration-dependent antioxidant and anticancer activities of CA and RA in ethanol extracts of three different Oregano species (*Origanum onites *L.,* Origanum vulgare* L., and* Origanum vulgare* ssp.* hirtum*). The study revealed the highest RA antioxidant activity in* O. vulgare *ssp.* hirtum* (9550 ± 95 mmol/g) and the lowest in* O. vulgare* L. (2605 ± 52 mmol/g) (*p* < 0.05). The highest CA amount was present in* O. onites* L., which was 1.8 and 4.7 times higher (*p* < 0.05) than in* O. vulgare* ssp.* hirtum *and* O*.* vulgare* L., respectively. The anticancer activity was evaluated on human glioblastoma (U87) and triple-negative breast cancer (MDA-MB231) cell lines* in vitro*. RA anticancer activity was negligible. CA and the extracts were about 1.5–2 times more active against MDA-MB231 cell line (*p* < 0.05) compared to U87 cell line. The anticancer activities of three tested extracts were similar against U87 cell line (*p* > 0.05) but they had different activities against MDA-MB231 cell line.

## 1. Introduction

There is a wide variety in nature of naturally occurring antioxidants that differ in their composition, physical and chemical properties, and action mechanisms [1]. Since the early work of Chipault et al., who have examined more than 70 spices and herbs, the interest in the antioxidant activity of spices has increased recently [2]. Since many of widely used synthetic antioxidants may cause negative health effects, herbs and spices could be the most important safe targets searching for the natural antioxidants [3]. Free radicals may disrupt normal or lead to the pathological cell metabolism causing diseases such as cancer, cirrhosis, and arteriosclerosis [4]. Natural antioxidants might play an important role in the free radical metabolism.

Many studies have demonstrated that spices and herbs that have high content of essential oils and phenolic compounds, such as Oregano, rosemary, and sage, serve as strong antioxidants [5]. In recent years, the efficiency of essential oils from Oregano species has been reported in many studies. Antioxidant activity of Oregano [6, 7] is associated with its constituents rosmarinic acid (RA) and carvacrol (CA). Oregano essential oils and phenolic compounds have been shown to possess antioxidant, anticancer, antibacterial, antifungal, diaphoretic, carminative, antispasmodic, and analgesic activities [8, 9]. Essential oils containing CA are the subject of increasing scientific interest because of their multiple biological effects, including antioxidant activity that is associated with the prevention of various degenerative diseases [9]. HPLC postcolumn assay is a highly promising method to investigate natural antioxidants with various derivatization techniques [10].

Some studies have used spectrophotometric method to determine a total antioxidant potency of Oregano [5, 11]. It is important that the antioxidant activity of the herb can be defined not only by the essential oil, but also by phenolic components or their interaction with other components [11]. Many studies have determined antioxidant activity of essential oils (EOs), but only few studies have been published on the antioxidant activity of individual components, for example, carvacrol and thymol [11–13]. A highly effective technique, developed and modified by different researchers, is based on a model oxidation system that combines HPLC separation methods with the online postcolumn detection of radical-scavenging compounds [14–16]. Online postcolumn methods are employed in the rapid identification of antioxidants and determination of their activity [10]. Model oxidation systems of DPPH radicals possess important advantages when compared with other colorimetric methods and thus have been extensively used in screening of plant extracts for antioxidants [17].

The objective of this study was to evaluate concentration-dependent antioxidant and anticancer activities of CA and RA in ethanol extracts of three different Oregano species (*Origanum onites *L.,* Origanum vulgare* L., and* Origanum vulgare* ssp.* hirtum*).

## 2. Materials and Methods

### 2.1. Plant Material

Dried* Origanum vulgare *L. herb was purchased from “Svencioniu zoles,” Lithuania; dried* Origanum vulgare *ssp.* hirtum *herb from “Çengelköy spice and herbs center,” Turkey; dried* Origanum onites *L. herb from “İnanTarım ECO DAB,” Turkey. Dried herbs were identified by Dr. Professor Jurga Bernatoniene, Medical Academy, Lithuanian University of Health Sciences. Identification of the plant material was performed according to the requirements of the European Pharmacopoeia, Monograph number 01/2008:1833 corr.6.0. The tests used include a pharmacognostical investigation of the anatomical and microscopical characteristics and TLC fingerprinting. Voucher specimens (number L170709, L170710, and L170711) have been deposited at the Herbarium of the Department of drug technology and social pharmacy, Lithuanian University of Health Sciences, Lithuania. The dried herb particle size of* O. vulgare *L.,* O. vulgare *ssp.* hirtum*, and* O. onites *was 125 *μ*m.

### 2.2. Chemicals

Extraction solvent ethanol (96%) was purchased from “Vilniaus degtinė” (Vilnius, Lithuania). Water used in HPLC and for the sample preparation was produced with a Super Purity Water System (Millipore, USA). HPLC eluents: methanol (99.95%) was purchased from Carl Roth GmbH (Karlsruhe, Germany) and acetic acid (99.8%) from Sigma-Aldrich (St. Louis, MO, USA). Standards for HPLC analysis: carvacrol (>98%) was purchased from Sigma-Aldrich (St. Louis, MO, USA) and rosmarinic acid (>98%) from ChromaDex (Santa Ana, TX, USA), respectively. 2,2-Diphenyl-1-picrylhydrazyl (DPPH, 95%) was purchased from “Sigma-Aldrich” (St. Louis, MO, USA). Trolox (98%) and TFA (99%) were received from “Fluka Chemika” (Buchs, Switzerland). Sodium citrate, citric acid, and potassium persulphate (99%) were purchased from “Sigma” (Sigma-Aldrich, Steinheim, Germany).

### 2.3. Preparation of Oregano Ethanol Extract

Prior to preparation of extract Oregano herb was ground in a cross beater mill IKA A11 Basic Grinder (IKA Works, Guangzhou, China) and sieved using vibratory sieve shaker AS 200 basic (Retch, UK) equipped with a 125 *μ*m sieve. Powdered material (100 g) was then extracted with 1000 mL of 90% (v/v) ethanol in a round bottom flask by heat-reflux extraction performed in the water bath Memmert WNB7 (Memmert GmbH & Co. KG, Schwabach, Germany) at 95°C for 4 hours. Prepared extract was filtered using vacuum filter. These conditions were determined in our previous study as the best for the extraction of main active compounds of Turkish Oregano [18].

### 2.4. HPLC Analyses

**Figure 1:**
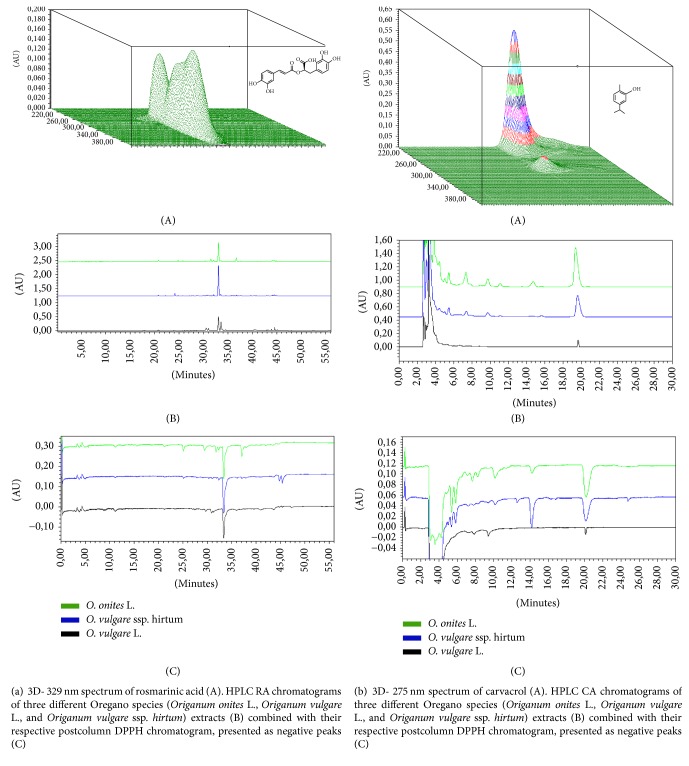


HPLC analysis has been carried out using Waters 2695 chromatography system (Waters, Milford, USA) equipped with Waters 996 PDA detector. Data was collected and analysed using a PC and the Empower2 chromatographic manager system (Waters Corporation, Milford, USA). For determination of RA ACE 5 C18 250 × 4.6 mm column (Advanced Chromatography Technologies, Aberdeen, Scotland) was used. Quantification of compounds was carried out by the external standard method. Standard stock solutions at a concentration of 1.0 mg/mL were prepared freshly in methanol and diluted in appropriate quantities to obtain a set of corresponding concentration ranges for the study of linearity. The regression coefficient (*R*^2^) was 0.999 for all the calibration curves. A calibration curve for each of the compounds was constructed by plotting peak areas versus the respective compound concentration and calculated by linear regression analysis. The precision of the method was demonstrated for all analytes, since all the obtained relative standard deviation (RSD) values were lower than 5.0%. The concentration of compounds was expressed as *μ*g/g dry mass (DM). HPLC conditions for determination of RA: the two elution solvents were exchanged: the solvent A (methanol) and the solvent B (0.5% (v/v) acetic acid in water). The following linear gradient elution profile was used: 95% A/5% B–0 min, 40% A/60% B–40 min, 10% A/90% B–41–55 min, and 95% A/5% B–56 min. The flow rate was 1 mL/min and injection volume was 10 *μ*L. The effluent was determined at a wavelength of 329 nm. The linear calibration curve was made and expressed by the following quadratic equation: *R*^2^  (RA) = 0.999918, (*y* = 2.01 · 10^7^*x* + 5.52 · 10^3^), the linearity range: 10–100 *μ*g/ml. (Figure 1(a)) [18]. For determination of CA: the mobile phase was composed of methanol and water (60/40, v/v). The flow rate was 0.6 mL/min and injection volume was 10 *μ*L. The absorbance was measured at 275 nm. The quantification has been carried out by the external standard method. The calibration curve was made and expressed by the following quadratic equation: *R*^2^  (CA) = 0.999751,  (*y* = 4.99*∗*10^9^*x* + 1.08*∗*10^3^), the linearity range: 0.18–3 *μ*g/ml (Figure 1(b)) [18].

### 2.5. HPLC Postcolumn Antioxidant Detection Method

According to Raudonis et al. [15], after applying the HPLC-PDA detection system, the mobile phase containing the analytes was entered into a reaction coil through a mixing tee where the reagents (DPPH solution) were supplied (split ratio, 1 : 1) at the same time by a Gilson pump 305 (Middleton, WI, USA). Reaction coils made of PEEK (Teflon) 10 m length, 0.25 mm i.d., and 1.58 mm o.d. were used (Waters PCR module, Milford, CT, USA). The system with DPPH solution was monitored as follows: temperature range was set at 50°C and the flow rate of the reagent was set at 1.0 mL/min for DPPH. DPPH solution was prepared following the instructions. The reaction of the antioxidant compounds with the DPPH reagent resulted in a colour change that was detected using an additional Waters 2487 UV/VIS detector (Waters Corporation). The detection of DPPH in solution was recorded at 520 nm wavelength. The signal strength, which is sensitivity related and reflected by the height of the negative peaks of the active compounds CA and RA, was chosen as the indicator for selecting analysis conditions. The postcolumn antioxidant activity of the extract compounds was assessed by comparing their activity to the standard, Trolox. Calibration curves were prepared from a Trolox ethanol solution at seven dilutions in the range of 0.02–0.12 mg/mL for DPPH. The calibration curves formed were equivalent to standard Trolox and were expressed by the following quadratic equation: *R*^2^  (DPPH) = 0.9973, (*y* = 1.95*∗*10^7^*x* + 6.46*∗*10^4^).

### 2.6. Cell Lines

Anticancer activity was tested on two selected cancer cell lines: human glioblastoma (U87) and human triple-negative mammary gland adenocarcinoma (MDA-MB231). Cells were cultured in Dulbecco's Modified Eagle's Medium (DMEM) high glucose (Gibco) supplemented with 10% fetal bovine serum (FBS) (Gibco) and 1% antibiotics (10000 units/mL penicillin and 10 mg/mL streptomycin) (Gibco) at 37°C in a humidified atmosphere containing 5% CO_2_. Cells were obtained from the American Type Culture Collection.

### 2.7. Determination of Anticancer Activity by MTT Assay

Cell viability was studied using the method of 3-(4,5-dimethylthiazol-2-yl)-2,5-diphenyltetrazolium bromide (MTT) (Sigma). 100 *μ*L of cells was seeded in 96-well plates in triplicate (5000 cells/well) and incubated at 37°C for 24 hours. Then serial dilutions of the pure compounds (CA and RA) or extracts were made in microplates. Medium without cells was used as a positive control. Cells treated with medium containing 0.25% dimethylsulfoxide (DMSO) (in the case of compounds) or ethanol (in the case of extracts) served as a negative control. After 72-hour incubation at 37°C, 10 *μ*L of MTT was added in each well. After 3 hours the liquid was aspirated from the wells and discarded. Formazan crystals were dissolved in 100 *μ*L of DMSO, and absorbance was measured at a test wavelength of 490 nm and a reference wavelength of 630 nm using a multidetection microplate reader. The experiments were repeated three times independently and the results were given as means ± SD.

### 2.8. Statistical Analysis

Statistical analysis was performed by one-way analysis of variance (ANOVA) followed by Tukey's multiple comparison test with the software package Prism v. 5.04 (GraphPad Software Inc., La Jolla, CA, USA). The value of *p* < 0.05 was taken as the level of significance.

## 3. Results and Discussion

**Figure 2:**
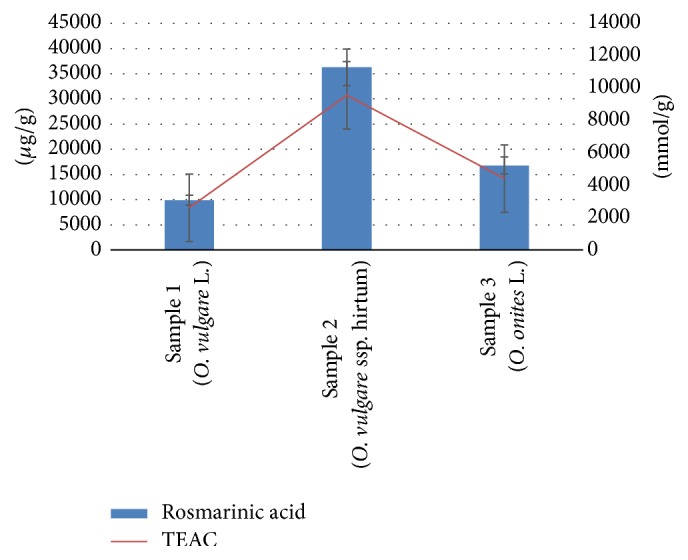
Concentration (*μ*g/g) and antioxidant activity (TEAC mmol/g) of RA from three different Oregano species (*Origanum onites* L.,* Origanum vulgare* L., and* Origanum vulgare* ssp.* hirtum*). Significant difference between all components *p* < 0.05.

**Figure 3:**
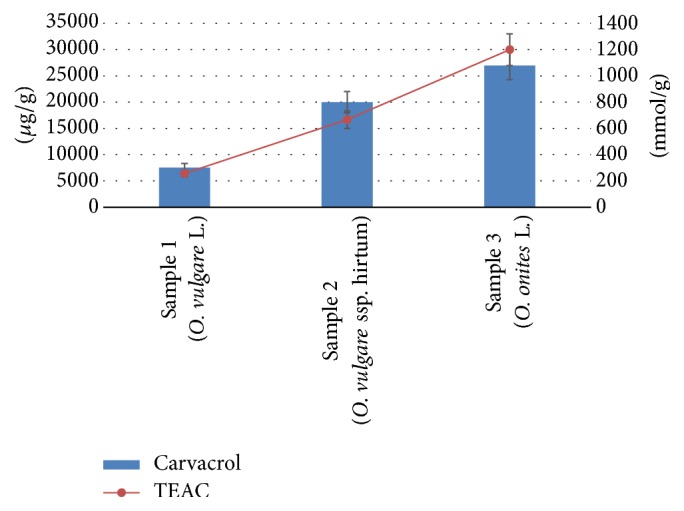
Concentration (*μ*g/g) and antioxidant activity (TEAC mmol/g) of CA from three different Oregano species (*Origanum onites* L.,* Origanum vulgare* L., and* Origanum vulgare* ssp.* hirtum*). Significant difference between all components *p* < 0.05.

In our previous study, we have used HPLC method for quantitative analysis of Oregano extract [16]. According to scientific literature the main active compounds in Oregano species herbs are phenolic compound-derived RA and CA from essential oil [4, 5]. Our results confirmed that the main compounds in all Oregano species were RA and CA (the chromatograms are shown in Figures 1(a) and 1(b)). The main differences between the species according to the amounts of RA and CA were shown in Figures 2 and 3. The highest yield of RA was revealed in *O*. ssp.* hirtum* L. and the highest yield of CA in* O. onites* L.

### 3.1. Antioxidant Activity

Phenolic compounds and the essential oil are the main sources of antioxidant activity [11]. The spectrophotometric method has been often used for the determination of antioxidant activity as total antioxidant activities [4, 9]. But still it is very difficult to obtain information on the contribution of individual compounds to the overall antioxidant effects due to complexity and diversity [19]. DPPH (2,2-diphenyl-1-picrylhydrazyl), a paramagnetic compound with an odd electron, is one of the most popular radicals used to evaluate antioxidant activities of pure substances and complex samples. HPLC postcolumn (bio) chemical assay determines activities of individual compounds present in the mixtures [20]. In the course of experimental investigations, HPLC-DPPH assays have been developed and applied for rapid screening and identification of antioxidants from the extracts of herbal medicines [21]. The HPLC-DPPH postcolumn methodology was used in further investigations of ethanol Oregano herb extracts of three different species and antioxidant activity of CA and RA has been determined.

The antioxidant activity of the measured RA compound has been expressed as TEAC values (Trolox mmol/g) (Figure 2). The results of DPPH postcolumn assay in terms of the TEAC values (mmol/g) for the measured RA compound showed statistically significant differences in antiradical response between three Oregano species. The highest antiradical response was obtained in* O. vulgare *ssp.* hirtum* (9550 ± 95 mmol/g) and the lowest in* O. vulgare* L. (2605 ± 52 mmol/g). Numerous studies have been published on total phenolic antioxidant activity of Oregano [11, 22]. Madsen et al. [23] reported that the antioxidant activity of Oregano is due to a variety of components, and a significant correlation between antioxidant activity and total phenolic content of the spice extract has been found [24].

Numerous studies have been published on antioxidant activity of essential oil of Oregano [4, 25, 26]. The present study proved the antioxidant activity of the identified CA compound from three different Oregano species and it was expressed as TEAC values (Trolox mmol/g). Results (Figure 3) showed statistically significant differences in the antioxidant activities of the identified CA compounds between three different Oregano species. The highest CA amount was obtained from* O*.* onites* L. While the concentration of CA was 1.8 times higher (*p* < 0.05) in* O*.* onites* L. compared with* O*.* vulgare* ssp.* hirtum*, the concentration of CA was 4.7 times higher (*p* < 0.05) in* O*.* onites* L. compared with* O*.* vulgare* L. (Figure 1(b)).* O. vulgare *accumulated the lowest amounts of CA and it led to the smallest values of TEAC.

The antioxidant activity of RA and CA as separate compounds has not been extensively studied yet to our knowledge. In our study, RA and CA have been tested for the first time in order to determine active components of the extract or essential oil of Oregano. Thus, our study puts importance on raising awareness about antioxidant properties of the specific components of Oregano. The results showed that the potency of antioxidant activity of Oregano depends on the species due to the different quantities of active substances (RA and CA) which is in good agreement with Lindberg Madsen report demonstrating that antioxidant activities are different for the different species [23].

### 3.2. Anticancer Activity

The anticancer activity of RA against tested cancer cell lines was negligible, even at the concentration of 50 *μ*M. Some other researchers [27, 28] also found that RA alone showed little effect on cancer cell viability.

**Figure 4:**
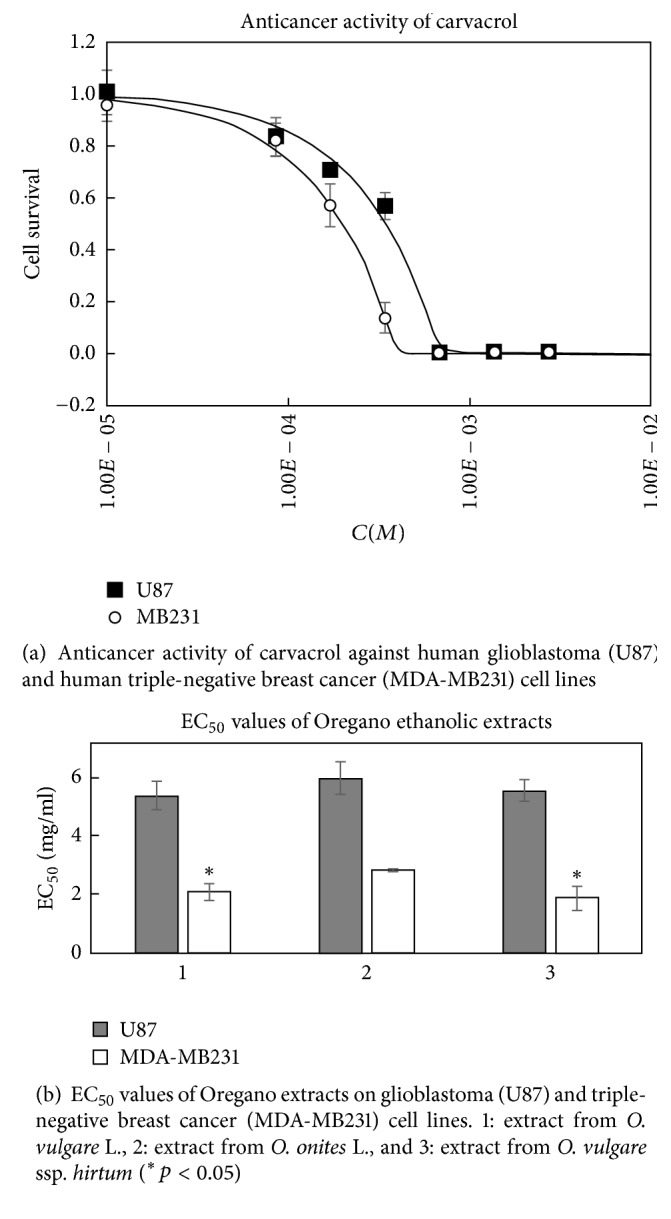


CA possessed 1.2 times higher activity (*p* < 0.05) against MDA-MB231 cells compared to U87 cells (Figure 4(a)). It inhibited the growth of 50% of both tested cancer cells at the concentrations of 199 *μ*M (MD-MB231) to 322 *μ*M (U87). The determined activity of CA is comparable with the results from other studies [29, 30].

All tested Oregano extracts showed antiproliferative activity* in vitro*. The extracts were about 2.1–2.9 times more active against MDA-MB231 cell line (*p* < 0.05) compared to U87 cell line (Figure 4(b)). This different activity could be due to the fact that glioblastoma cells are usually more resistant to chemo- and radiotherapy; they may contain accumulated gene mutations and epigenetic alterations [31].

**Figure 5:**
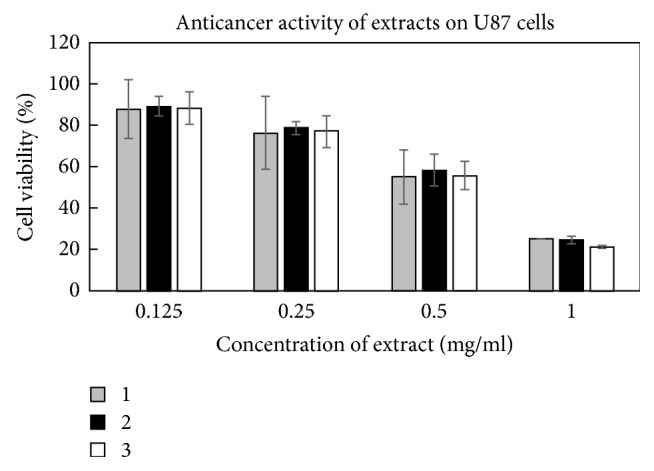
Activity of Oregano extracts against glioblastoma (U87) cell line. 1: extract from* O. vulgare* L., 2: extract from* O. onites* L., and 3: extract from* O. vulgare* ssp.* hirtum*.

**Figure 6:**
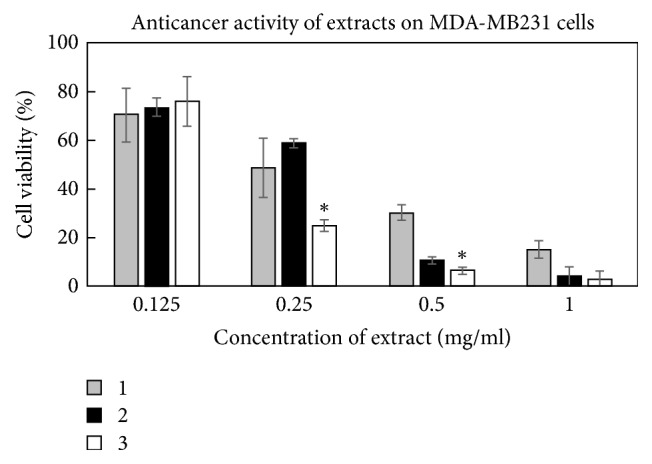
Activity of Oregano extracts against triple-negative breast cancer cell line MDA-MB231. 1: extract from* O. vulgare* L., 2: extract from* O. onites* L., and 3: extract from* O. vulgare* ssp.* hirtum* (^*∗*^*p* < 0.05).

The anticancer activities of all three tested extracts were very similar against U87 cell line (*p* > 0.05) (Figure 5), except the different activity against MDA-MB231 cell line (Figure 6). The extracts prepared from* O. vulgare* ssp.* hirtum* and* O. vulgare* L. were more active (*p* < 0.05; EC_50_ values were 2.0 ± 0.3 mg/mL and 1.8 ± 0.4 mg/mL, resp.). Interestingly, the extracts possessed different activities on MDA-MB231 cell line depending on their concentration (Figure 6). Extract prepared from* O. onites* L. had lower activity at the concentration of 0.25 mg/mL but was similarly active as the extract prepared from* O. vulgare* ssp.* hirtum *at the highest tested concentration of 1 mg/mL. Those different species contain different quantities of active substances and thus could possess different antiproliferative activities against the same cancer cell line [32].

## 4. Conclusion

The selective HPLC-DPPH postcolumn methodology was used to identify CA and RA in ethanol extract of three different Oregano (O.* onites* L., O.* vulgare *L., and O.* vulgare *ssp.* hirtum* L.) species. Differently from other studies showing that the crude extracts of Oregano possess antioxidant activity, our experiments clearly demonstrate the antioxidant activities of RA and CA as separate compounds.

Our investigations revealed that RA is an efficient natural antioxidant and it possesses higher antioxidant activity than CA in all species of Oregano. In contrast to* O*.* onites*, in* O. vulgare *L. and O.* vulgare *ssp.* hirtum* L. the antioxidant activity depended on the amount of raw material and potency of antioxidant activity of CA.

As a final conclusion of this study, the potency of antioxidant activity of Oregano varies in the species due to different quantities of RA and CA. The anticancer activity of the tested extracts was higher against human triple-negative breast cancer cell line compared to glioblastoma cell line. The anticancer activity of Oregano extracts depends on both the tested cell line and the species.
